# The Ways of Isolating Neoantigen-Specific T Cells

**DOI:** 10.3389/fonc.2020.01347

**Published:** 2020-08-11

**Authors:** Qing Li, Zhen-Yu Ding

**Affiliations:** Department of Biotherapy, Cancer Center, National Clinical Research Center for Geriatrics, West China Hospital, Sichuan University, Chengdu, China

**Keywords:** adoptive cell therapy (ACT), neoantigen-specific T cells, T cell receptor (TCR), tumor infiltrating lymphocytes (TILs), peripheral blood lymphocytes (PBLs)

## Abstract

Immunotherapy has revolutionized the standard of care for a range of malignancies. Accumulating evidence suggests that the success of immunotherapy is likely attributable to neoantigen-specific T cells. Thus, adoptive cell therapy with these neoantigen-specific T cells is highly promising. This strategy has proven to successfully elicit tumor regression or even complete remission in metastatic cancer patients. However, a fundamental challenge is to effectively identify and isolate neoantigen-specific T cells or their T cell receptors (TCRs), from either tumor-infiltrating lymphocytes (TILs) or peripheral blood lymphocytes (PBLs), and many methods have been developed to this end. In this review, we focus on the current proposed strategies for identifying and isolating neoantigen-specific T cells.

## Introduction

Several immunotherapeutic strategies that harness the exquisite specificity of the immune system to eliminate tumors have emerged during the past decade; these include cancer vaccines, immune checkpoint blockade, and adoptive cell therapy (ACT) (1) with the potential to revolutionize the standard of care for a range of malignancies.

To a large extent, the specificity of immunotherapy is dependent on the recognition of specific tumor antigens, especially neoantigens. Neoantigens are a kind of tumor antigen derived from tumor-specific somatic mutations and are highly restricted to tumor cells with minimal established immune tolerance (1). Neoantigen-based cancer vaccines have shown promising therapeutic effects in the clinic (2–8). In addition, a growing body of evidence indicates that neoantigen-specific T cells underlie the success of the recently emergent immune checkpoint inhibitor therapy (9–13). Adoptive transfer of autologous, *in vitro* expanded, tumor-infiltrating lymphocytes (TILs) was reported to achieve dramatic clinical responses in some metastatic cancer patients, especially in those with melanoma and cervical cancer (14–19). In-depth studies have revealed the critical roles of neoantigen-specific T cells in maintaining durable responses following ACT (20–26). In support of these findings, the adoptive transfer of selected TILs targeting neoantigens led to significant tumor regression (27–29). Increasing research attention has been shifted to identifying and selecting neoantigen-specific T cells (30–34). However, such a “precise targeting” strategy poses a great challenge in terms of the identification and isolation of neoantigen-specific T cells. Methods have been proposed and developed for this purpose. Here, we attempt to summarize the known strategies for isolating neoantigen-specific T cells.

## Identification and Isolation of Neoantigen-Specific T Cells From TILs

Researchers have long attempted to isolate neoantigen-specific subpopulations from the background of transferred TILs. In early studies, an autologous tumor cell cDNA library was constructed and used as a pool to screen for neoantigen-specific T cells (20, 21). In a study of a melanoma patient who experienced a complete response going beyond 7 years following adoptive TIL transfer, one T cell clone specific for a mutated antigen PPP1R3B was identified and shown to be responsible for the antitumor effects (22).

However, the time-consuming and laborious process required to identify neoepitope-responsive T cells has hindered their extensive functional assessment (32). Advances in next-generation sequencing have enabled the rapid assessment of the mutational landscape of human cancers and made it possible to identify immunogenic mutated tumor antigens through *in silico* analysis. Rosenberg's group first employed predicted neo-peptides, obtained by whole-exome sequencing and human leucocyte antigen (HLA) class I–binding algorithms, for TIL screening. Using this approach, they identified 7 neoantigens recognized by 3 therapeutic bulk TIL cultures that mediated objective tumor regressions in three individuals with melanoma (23). Using a similar method, neoantigen-specific CD8+ TILs could also be identified in hematological malignancies, such as acute lymphoblastic leukemia (ALL) (35). Prickett et al. (25) and Stevanovic et al. (26) also demonstrated that neoantigen-specific T cells could be identified from therapeutic TILs by screening tandem minigene (TMG) libraries encoding cancer mutations identified from patients' tumors by whole-exome sequencing. This finding might further facilitate the recognition of neoantigen-specific T cells because it circumvents the need for prediction of HLA–peptide binding and synthesis of a large number of peptides.

**Figure 1:**
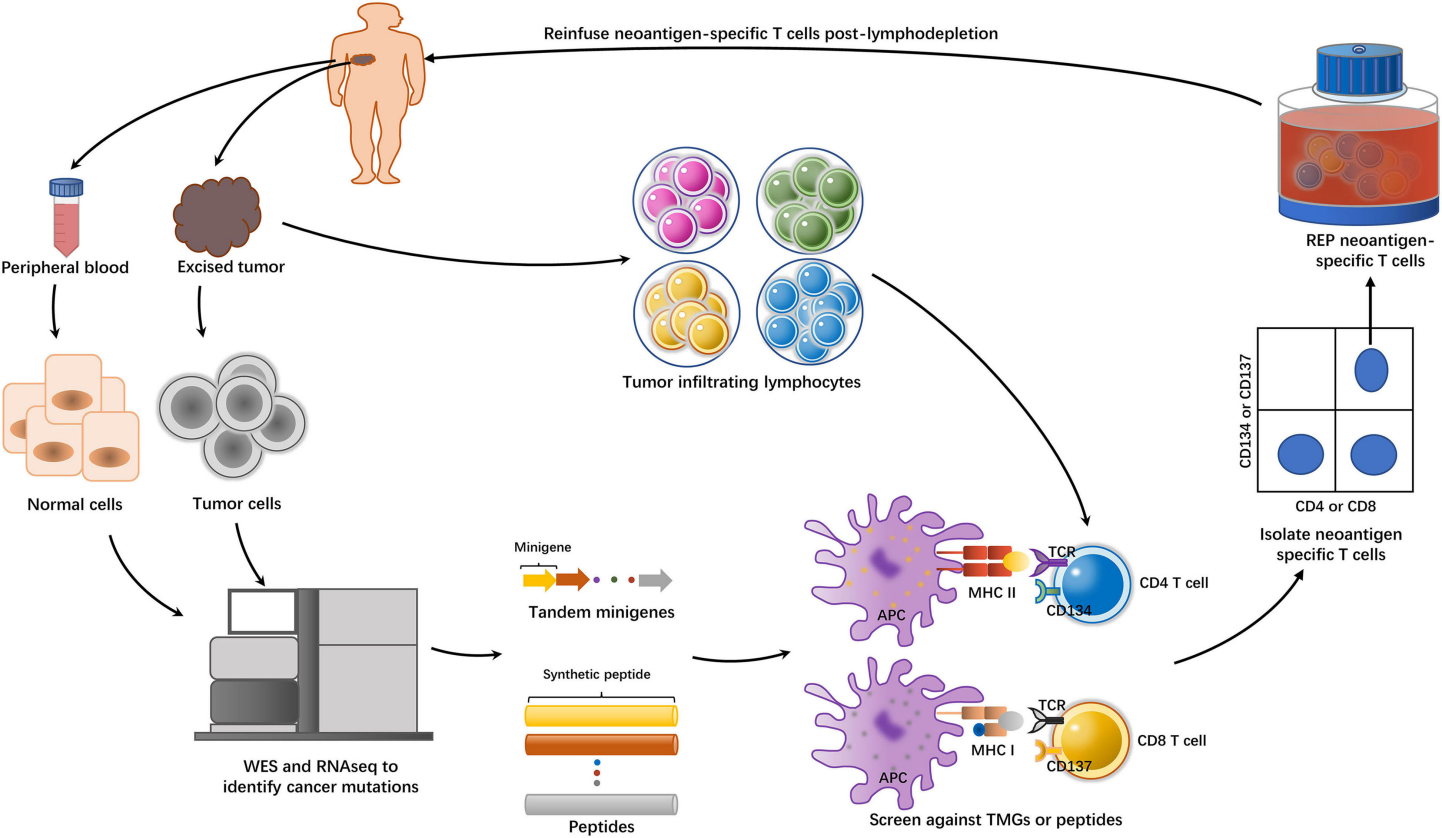
The general approach of identifying and isolating neoantigen-specific TILs for ACT. The tumor cells from excised tumor tissue and matched normal cells underwent whole-exome sequencing (WES) and RNA sequencing to identify non-synonymous mutations. Based on the information, either tandem minigenes (TMGs) or peptides were then synthesized. These TMGs or peptides were pulsed into autologous antigen presenting cells (APCs), such as dendritic cells (DCs) or B cells, and they were processed and presented in the context of major histocompatibility complex (MHC). On the other side, the excised tumors were minced into ~1–2 mm^3^ fragments and placed in 24-well plates stimulated with IL-2. Then, the TILs were cocultured with these pulsed APCs. The identification of the individual neoantigen-specific T subpopulation was dependent on the IFN-γ enzyme-linked immunospot (ELISPOT) assay and the activation of the markers such as CD137(41BB) or CD134(OX40) on the T cell surfaces when recognizing their cognate target antigen. T cells with these activation surface markers would be purified by flow cytometry. Then, the sorted T cells were subject to rapid expansion *in vitro* and reinfusion to the tumor-bearing patient.

With the advent of these techniques, the field of ACT took a great leap from bulk TILs to neoantigen-specific T cells. A concise flowchart showing the steps involved in identifying and isolating neoantigen-specific T cells for ACT is summarized in Figure 1. Tran et al. (27) successfully performed neoantigen-specific T cell therapy in a 43-year-old woman with extensively metastatic and intensively treated cholangiocarcinoma. After administration of a bulk lymphocyte population containing a high percentage of neoantigen ERBB2IP-specific CD4+T cells, the patient showed a long-lasting objective clinical response without obvious toxicity. Subsequently, neoantigen-specific T cells were identified in one colon cancer patient and another breast cancer patient, and reinfusion of these specific T cells led to a partial response in one patient and a durable complete response in another (28, 29). Currently, ACT with neoantigen-specific T cells is being tested in clinical trials in both solid and hematological tumors (Supplementary Table 1).

However, the extensive expansion of neoantigen-specific T cells during preparation compromises their proliferation potential (36). In addition, the method involved requires sophisticated equipment and a time period of several months. For most metastatic patients, this time frame is unacceptable. To address these issues, additional attempts have been made, using either surface markers or T cell receptor (TCR) redundancy.

## Approaches Based on Surface Markers

CD137 belongs to the tumor necrosis factor receptor superfamily (37, 38). It functions as a costimulatory molecule to promote the proliferation and survival of activated T cells (39, 40). CD137 expression is highly restricted to transiently activated CD8+ T cells but almost undetectable in resting cells. Upregulated CD137 can be detected on stimulated CD8+ T cells of all phenotypes (e.g., naïve T cells as well as early and late memory effector T cells) (41). Naturally occurring tumor-reactive T cells stimulated by tumor antigens also express CD137 as proven by Ye et al. (42). In a clinical trial (Trial registration ID: NCT02111863) among 6 patients with melanoma who underwent adoptive transfer with CD137-selected TILs, only 1 patient achieved partial response, and the remaining 5 progressed. The study was terminated.

This approach has its pitfalls: Because CD137 is an activation marker, CD137+ T cells obtained by large-scale production are generally overactivated and highly differentiated with limited proliferative potential. A potential solution is to obtain TCRs from these CD137+T cells instead. This strategy was reported by Parkhurst et al. (43). Briefly, CD8+ T cells were stimulated overnight with immunogenic mutated TMG RNAs. Subsequently, the CD8+ T cell population with the highest CD137 expression was sorted by fluorescent-activated cell sorting (FACS), and expanded *in vitro*. Then, dominant TCR α and β chains were sequenced in the enriched populations. Twenty-seven TCRs from 6 patients that recognized 14 neoantigens expressed by autologous tumor cells were identified. However, this process was time-consuming (2–3 months).

A simplified protocol was proposed by Seliktar-Ofir et al. (44). Here, TILs, but not CD8+ T cells, were cocultured with autologous tumor cells; CD137+ T cells were isolated by magnetic bead separation and expanded. No further TCR sequencing was performed. The entire process took only 35 days. T cells stimulated with neoantigens or other tumor-associated antigens exhibit upregulated CD137 expression (25, 42, 43, 45). Therefore, a CD137-based selection protocol was advocated for its broad antigen coverage including both neoantigen and shared tumor antigens without prior knowledge of epitope specificity. However, the prerequisite of the establishment of autologous tumor cell lines poses a challenge.

Direct and indirect evidence shows that the interaction between PD-1 and PD-L1 inhibits T lymphocyte function, leading to evasion of persistent inflammatory or autoimmune reactions (46–48). However, this protective mechanism is hijacked by tumors to escape immune surveillance, PD-1 has been characterized as an inhibitory receptor on chronically stimulated T-cells in the tumor microenvironment (46). At the tumor site, TILs are exposed to tumor antigens; the binding of TCR and antigen upregulates either costimulatory or coinhibitory receptors to promote or inhibit T cell activation and function, respectively (49). Therefore, PD-1+ T cell populations among TILs may contain a large proportion of tumor-specific T cells. The findings of Inozume et al. (50) and Ahmadzadeh et al. (51), that tumor-responsive T cells are enriched among CD8+PD1+ lymphocytes from fresh melanoma specimens, provide direct support for this notion.

In another study, Gros et al. (49) demonstrated that PD-1 expression on CD8+ TILs in fresh melanoma tumor specimens enabled identification of a diverse patient-specific repertoire of clonally expanded tumor-reactive cells, including mutated neoantigen-specific CD8+ lymphocytes. Although PD-1 is an inhibitory receptor expressed on T cells, studies have shown that IL-2 restored the antitumor function of T cells *in vitro* (49, 50). However, on antigen-experienced terminally differentiated effector memory (T_EMRA_) cells, PD-1 is either not expressed or expressed at very low levels (49, 52). Therefore, a PD-1-based enrichment strategy may not be suitable for these cells.

Screening strategies based on CD137 or PD-1 expression are suitable for CD8+ T cells, mainly in melanoma. Epithelial cancers, which account for more than 80% of all human malignancies, harbor fewer mutations than melanoma (53). They exhibit compromised capability to induce mutation-specific T cell responses, together with a limited number of infiltrating neoantigen-specific TILs (32). In addition, CD4+T cells have been shown to play an important role in mediating tumor regression in animal models and patients (27, 36, 54–56). However, CD137 or PD-1 is expressed on CD8+ cells as a sole marker; therefore, it may not be reliably used to enrich activated CD4+ cells (42, 43, 57). CD134 is transiently expressed on CD4 + T cells stimulated by antigens and can be used as a marker for the classification of mutant reactive T cells (58).

Recently, Yossef et al. (36) reported an approach in which the TILs that expressed CD134 or CD137and/or PD-1 were isolated by FACS. Thus, both CD4+ T and T_EMRA_ cells were rescued, which would otherwise be missed if a single marker were used. Sorted cells underwent limiting-dilution in microwell plates to avoid the overgrowth of non-specific T cells. Cultures were tested for the ability to recognize a 25-mer peptide pool encompassing possible neoantigens. Notably, the highly oligoclonal nature of these T cells makes possible the convenient application of single cell sequencing of their TCRs. In 6 patients with metastatic epithelial cancer, this high-throughput approach led to the detection of CD4+ and CD8+ T cells targeting 18 and 1 neoantigens, respectively, whereas only 6 and 2 neoantigens were identified by using the TIL fragment screening approach. In 2 patients in which no neoantigen was found by traditional screening, the novel approach identified 5 distinct neoantigen-specific TCR clones for one patient and a highly potent MHC class II–restricted KRAS^G12V^-reactive TCR for the other. In a metastatic tumor sample from a patient with serous ovarian cancer, 3 MHC class II–restricted TCRs targeting the TP53^G245S^ hot-spot mutation were identified.

## TCR Frequency

TCR sequence analysis is used as a tool to monitor T cell responses to specific antigens by measuring the abundance of T cell clonotypes (49, 59, 60). The advent of next-generation sequencing has enabled identification of the full TCR repertoire of TILs (61, 62). This valuable data for TCRs from tumor-reactive TILs could be used to modify T cells (TCR-T). However, the lengthy expansion process and excessive stimulation would result in TCR repertoire switching (63). To avoid this problem, Pasetto et al. (63) directly performed TCR sequencing of the fresh enzymatically digested melanoma tissues prior to *in vitro* expansion. As described earlier, tumor-reactive clonotypes were enriched in CD8+PD-1+ TIL subsets in melanoma (49, 50). The authors analyzed the TCR repertoire of TILs in CD8+, CD8–, CD8+PD-1–, or CD8+PD-1+ subsets, respectively, and found that many of the most frequently occurring TCR clonotypes present in the CD8+PD-1+ TIL subset recognized the autologous tumor and tumor antigens, included neoantigens. This report provided a much more convenient approach to efficiently identify tumor-reactive T cells based solely on the frequency of TCR and PD-1 expression, without prior knowledge of the specific neoantigen. However, this strategy must be applied with caution because the isolated TCR clones may be self-reactive and result in deleterious on-target, off-tumor toxicities (43).

## Isolation of Neoantigen-Specific T Cells From Peripheral Blood Lymphocytes (PBLs)

In some situations, neoantigen-specific T cells were undetectable in the TIL compartment, possibly owing to the following factors: presentation of neoantigens in a non-inflammatory context (64), impaired T cell infiltration because of the sparse distribution of adhesion molecules on these cells (65, 66), and presence of immunosuppressive cytokines and cells (e.g., regulatory T cells) in the tumor microenvironment (67). Furthermore, the tissue from which TILs may be obtained poses a challenge. In this regard, peripheral blood is an alternative and reliable source for neoantigen-specific T cells.

The first attempt is considered to have been made by a group led by Lennerz et al. (68). In this study, a system of “mixed lymphocyte-tumor cells” (MLTC) was established, wherein peripheral blood mononuclear cells (PBMCs) from a patient with metastatic melanoma were cocultured with autologous tumor cells. The MTLC system could be viewed as a simplified *in vitro* simulation of the tumor microenvironment. Furthermore, cytotoxic T lymphocyte (CTLs) clone derived by limiting dilution from the MLTC system or MLTC were subject to autologous tumor cell cDNA library screening. T cell clones reactive to 5 mutated epitopes were obtained.

The use of MHC-peptide tetramers is a canonical method to identify and study a certain antigen-specific T cell subset (69–71). For ACT, tetramers were used to isolate and expand tumor antigen-specific T cells (72). Moreover, in immune checkpoint inhibitor (ICI)-treated cancer patients, MHC-peptide tetramers have been successfully used to monitor neoantigen-specific T cells (9, 12). Cohen et al. (73) used this method to sort neoantigen-specific T cells from the PBLs of patients with metastatic melanoma. In brief, a panel of MHC-peptide tetramers consisting of predicted neo-epitopes was synthesized and used to screen PBLs. Neoantigen-specific T cells targeting 8 of the 9 mutated epitopes identified from TILs could be isolated from autologous peripheral blood with frequencies ranging between 0.4 and 0.002%. In cancers with intermediate mutational loads, such as multiple myeloma, the use of MHC-peptide tetramers could also isolate neoantigen-specific T cells from the PBLs (74). However, this method was only applied to CD8+ T cells and required HLA-binding prediction algorithms to guide the synthesis of HLA-peptide tetramers.

A previous study has shown that PD-1 expression could guide the identification of neoantigen-specific CD8+ T cells from the tumor microenvironment (49). The same strategy could be adopted for isolation from PBLs (75). In one study, 4 patients with metastatic melanoma were enrolled. CD8+ PBLs were expanded *in vitro* and cocultured with autologous DCs, which were electroporated with *in vitro* transcribed TMG RNA for mutant epitopes. In 3 out of 4 patients, neoantigen-specific lymphocytes could be isolated from the CD8+PD-1+ lymphocyte subset, but not the CD8+PD-1– lymphocyte subset (75).

The isolation of neoantigen-specific cells from the PBLs of patients with epithelial cancer is even more challenging. Preexisting antigen-specific memory T cells may represent a potential solution. Memory T cells, including central memory T cells (T_CM_), effector memory T cells (T_EM_), and T_EMRA_ from PBLs were cocultured with DCs loaded with candidate neoantigens in the TMG or peptide form (76). After coculturing, memory cells were restimulated with DCs loaded with all TMGs and then sorted by the expression of CD134 and CD137 to enrich for neoantigen-reactive T cells. The resulting cells were then expanded and screened against all TMGs to test for neoantigen recognition. With this highly sensitive “*in vitro* stimulation (IVS)” method, T cells targeting KRAS^*G*12*D*^ and KRAS^*G*12*V*^ were successfully isolated from 3 out of 6 epithelial cancer patients. This new method enabled identification and isolation of neoantigen-reactive T cells from the blood circulation at very low frequencies.

The identification of neoantigen-specific T cells from naïve T cells is also of interest. A previous report showed that both naïve and activated neoantigen-specific T cells could be expanded from the peripheral blood of follicular lymphoma patients by priming with peptide-pulsed DCs (77). Using the same method, neoantigen-specific T cells were successfully expanded from the peripheral blood of HLA-matched healthy donors (30, 78). These preliminary results support the use of naïve T cells as an alternative source for ACT; however, their exceptionally low frequencies in peripheral blood and requirement for repeated stimulation pose hurdles (79).

Recently, a large, library-based “mini-lines” screening approach was proposed, which aimed to identify naïve antigen-reactive T cells from small volumes of blood (80–82). This system began with a small-scale culture in 96-well plates with 2,000 initial T cells in each well. The small-scale culture underwent a rapid 1,000- to 5,000-fold expansion (mini-line). Thousands of such well-scaled cultures were conducted simultaneously. Each T cell clone was maintained at a frequency of 1 in 2,000 but amplified to an absolute number of 1,000–5,000 cells, which is a sufficient number for routine detection. Applying this high-throughput parallel T cell culture system, neoantigen-specific T cells were identified and expanded 3–9 months prior to the first tumor recurrence in a patient with high-grade serous ovarian cancer. However, the long duration of culture possibly rendered this method more suitable as a preemptive therapeutic strategy (83).

## Discussion

**Figure 2:**
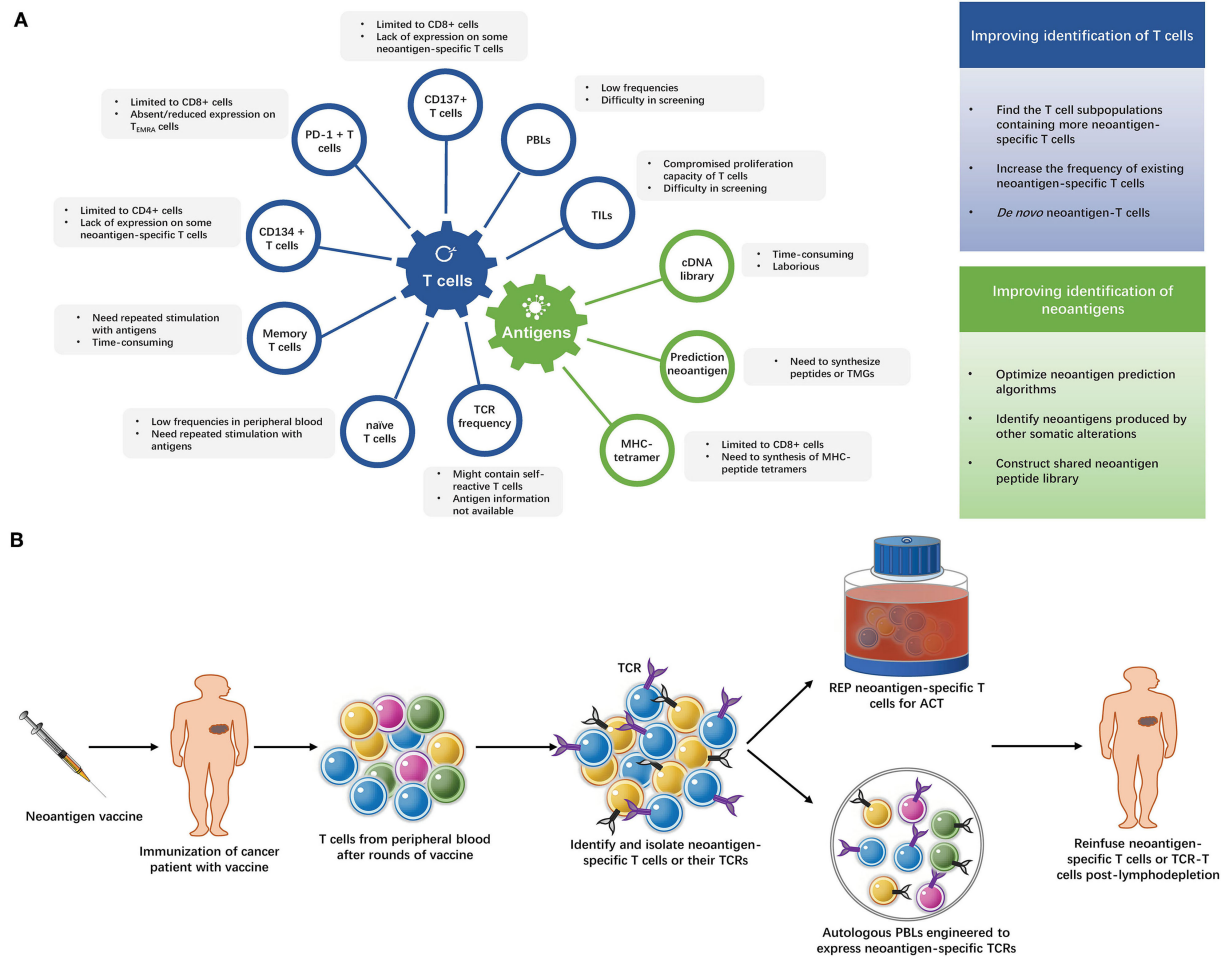
**(A)** Strategies of identifying neoantigen-specific T cells. The limitations of current methods of identifying neoantigen-specific T cells and strategies to improve neoantigen-specific T cells identification. TILs, tumor infiltrating lymphocytes; PBLs, peripheral blood lymphocytes; PD-1, programmed cell death-1; T_EMRA_ cells, terminally differentiated effector memory cells; TCR, T cell receptor; TMG, tandem minigene; MHC, major histocompatibility complex. **(B)** The “blueprint” of isolating neoantigen-specific T cells from peripheral blood after neoantigen-targeting vaccine. After several rounds of immunization with neoantigen vaccines, T cells are collected from the patient's peripheral blood and neoantigen-specific T cells are identified and isolated from these T cells. Then, the neoantigen-specific T cells undergo rapid expansion (REP), or their TCRs are exploited to modify autologous lymphocytes. The expanded neoantigen-specific T cells or modified TCR-T cells are reinfused to the patient.

After decades of efforts, the adoptive transfer of neoantigen-specific T cells is finally close to readiness for clinical application. High efficacy of this immunotherapeutic strategy has been achieved in a number of cancer patients and the prospects are promising. However, these approaches are also quite costly and hard to apply to large numbers of patients. The current methods of identifying neoantigen-specific T cells are summarized in Figure 2A and Supplementary Table 2. More convenient and effective screening methods for neoantigen-specific T cells remain necessary, some strategies to improve neoantigen-specific T cells identification are shown in Figure 2A.

It is feasible to obtain neoantigen-targeting T cells from PBLs although their frequencies are generally much lower than TILs (73, 75, 76). However, increasing the frequencies of these valuable neoantigen-specific T cells in peripheral blood remains a challenge.

Vaccination with neo-peptides has been shown to prime CD4+ and CD8+ T-cell responses in mouse models (54, 84). Patients treated with vaccines generated neoantigen-specific T cells (2–8). It could be reasonably inferred that the isolation of neoantigen-reactive T cells from the peripheral blood would be more easily achieved following neoantigen-specific vaccination. This neoantigen-based combo immunotherapy has its advantages: first, isolation and expansion of TILs *in vitro* is not necessary. Second, cancer vaccines not only elicit neoantigen-specific T cell responses and amplify existing tumor-specific T cells responses, but they also increase the breadth and diversity of the tumor-specific T cell response (1, 7). Multiclonal T cells may, thus, be obtained. Third, the relatively easy preparation of cancer vaccines would buy time for the isolation of neoantigen-specific T cells in maintaining the performance of patients. The “blueprint” is shown in Figure 2B.

## Conclusion

The previous decade has witnessed the emergence of immunotherapy for cancer. Accumulating evidence suggests that neoantigen-specific T cells underlie successful immunotherapy. Therefore, the isolation of neoantigen-specific T lymphocytes represents the “holy grail” for cancer immunotherapy. However, a fundamental challenge is to effectively identify and isolate neoantigen-specific T cells. The developments summarized in this review and future breakthroughs are anticipated to translate the adoptive transfer of neoantigen-specific T cells into a powerful weapon in our armamentarium against cancer.

## Author Contributions

QL prepared the manuscript draft. Z-YD revised it critically for important intellectual content and approved the final version. QL and Z-YD contributed to the conception and design of the review. All authors contributed to the article and approved the submitted version.

## Conflict of Interest

The authors declare that the research was conducted in the absence of any commercial or financial relationships that could be construed as a potential conflict of interest.
