# Supplementary material for: The Ways of Isolating Neoantigen-Specific T Cells
Source: Front Oncol. 2020 Aug 11;10:1347. doi: 10.3389/fonc.2020.01347 (PMC7431921; doi:10.3389/fonc.2020.01347)
Supplement: Supplementary file 1 [file Table_1.DOCX]

**Supplementary Table 1. Ongoing clinical trials registered in ClinicalTrials.gov investigating neoantigen-specific T cells/TCR-based ACT for the treatment of human cancer.**

| **Clinical Trials** | **Objective** | **Approach** | **Tumor Type** | **Phase** | **Status** |
| --- | --- | --- | --- | --- | --- |
| NCT03658785 | Evaluate the safety, side effects and benefits of autologous TILs specific to personalized neoantigens in the treatment of patients with recurrent, metastatic and advanced solid tumors. | Neoantigen-specific TILs | Recurrence tumor  Metastatic cancer  Solid tumor | Phase 1  Phase 2 | Not yet recruiting |
| NCT03171220 | Study the safety and efficient of neoantigen reactive T cells (NRTs) combined with programmed cell death-1(PD-1) inhibitor (SHR-1210) in the treatment of Chinese patients with advanced refractory solid tumors. | NRTs combined with SHR-1210 | Advanced malignant solid tumor | Phase 1  Phase 2 | Recruiting |
| NCT02632019 | Evaluate the safety and prognosis of dendritic cell-precision T cell for neo-antigen (DC-PNAT) in the treatment of advanced biliary tract malignant tumor. | Gemcitabine combined with or without DC-PNAT | Advanced biliary tract malignant tumor | Phase 1  Phase 2 | Unknown status |
| NCT03258359 | Evaluate the safety of personalized adoptive cell therapy targeting MDS stem cell neoantigens (PACTN). | PACTN | Myelodysplastic syndromes | Phase 1 | Recruiting |
| NCT02959905 | Evaluate the safety of tumor specific antigen-induced cytotoxic T lymphocytes (TSA-CTL) in the treatment of the advanced solid tumor. | TSA-CTL | Advanced malignant solid tumor | Phase 1 | Recruiting |
| NCT03970382 | Study the safety, feasibility, and efficacy of a single dose of neoantigen TCR-T cells (NeoTCR-P1) or in combination with anti-PD-1 in participants with locally advanced or metastatic solid tumors. | NeoTCR-P1 with or without Anti-PD1 | Melanoma  Urothelial carcinoma  Ovarian cancer  Colorectal cancer  Breast cancer (HR+)  Prostate cancer | Phase 1 | Not yet recruiting |
| NCT03412877 | Study the efficacy of genetically engineered TCR-T cells reactive against mutated neoantigens in patients with certain metastatic cancer | TCR-T cells reactive against mutated neoantigens | Glioblastoma  Non-small cell lung cancer  Ovarian cancer  Breast cancer  Gastrointestinal/Genitourinary cancer | Phase 2 | Recruiting |

TILs, tumor infiltrating lymphocytes; NRTs, neoantigen reactive T cells; DC-PNAT, dendritic cell-precision T cell for neo-antigen; PACTN, personalized adoptive cellular therapy targeting myelodysplastic syndromes (MDS) stem cell neoantigens; TSA-CTL, tumor specific antigen-induced cytotoxic T lymphocytes; NeoTCR-P1, gene edited neoantigen targeted T cell receptor modified T cell; HR, hormone receptor; TCR-T, T cell receptor modified T cell.
