# Supplementary material for: The Ways of Isolating Neoantigen-Specific T Cells
Source: Front Oncol. 2020 Aug 11;10:1347. doi: 10.3389/fonc.2020.01347 (PMC7431921; doi:10.3389/fonc.2020.01347)
Supplement: Supplementary file 2 [file Table_2.DOCX]

**Supplementary Table 2. Selected trials of identifying neoantigen-specific T cells or their TCRs.**

| **Author (Reference number)** | **Tumor histology** | **No. of patient(s)** | **No. of patient(s) found neoantigen-specific T cells** | **No. of neoantigen(s)** | **Target neoantigen(s)** | **T cell type** | **No. of reactive TCRs found** | **Comments** |
| --- | --- | --- | --- | --- | --- | --- | --- | --- |
| **Identification and isolation of neoantigen-specific T cells/TCR from TILs** | | | | | | | | |
| Robbins (22) | Melanoma | 1 | 1 | 1 | PPP1R3B | / | / | Identify neoantigen-specific TILs by screening of autologous tumor cell cDNA library using the therapeutic TILs in a patient with melanoma who experienced a complete response following ACT |
| Lu  (23) | Melanoma | 3 | 3 | 7 | CSNK1A1; GAS7; HAUS3; PLEKHM2; PPP1R3B; MATN2; CDK12 | / | / | The first study to use therapeutic TIL cultures screening of candidate mutated epitopes obtained from whole-exome sequencing (WES) of autologous tumor |
| Prickett  (24) | Melanoma | 2 | 2 | 2 | KIF2C; POLA2 | / | / | Identify neoantigen-specific TILs by screening autologous antigen-presenting cells that were transfected with TMGs |
| Tran  (27) | Cholangiocarcinoma | 1 | 1 | 1 | ERBB2IP | CD4 | 3 | The first study of using neoantigen- specific T cells for ACT |
| Parkhurst  (42) | Melanoma | 7 | 6 | 14 | COL18A1; SRPX; KIF16B; TFDP2; UGGT2; XPNPEP1; KIAA1279; KIAA1967; PHKA1; SON; GNB5; FBXO21; CORO7; RECQL5 | CD8 | 27 | Isolation of neoantigen-specific TCR from TILs based on CD137 expression |
| Yossef  (35) | Epithelial cancer | 6 | 6 | 19 | GBASE207K; PLXNB3; DLAT; TMPRSS4; PSMD2 HIST1H1B; HYAL4; HSPG2; KRAS^G12V^; MAP3K2 UEVLD-1/2; RAD51B; TBCK; TP53; HIST1H2BM; GORASP; TUBA1B; ZNF727TNC | CD4:18  CD8:1 | 36 | Enhanced detection of neoantigen-specific TILs by enriching T cells that express PD-1 and/or T cell activation markers followed by microwell culturing |
| Pasetto  (62) | Melanoma | 10 | 5 | 7 | HLA-A11; FBXO21; HELZ2; SRPX; WDR46; KIAA-iso; MAGEA6 | CD8 | 11 | Isolation of neoantigen-specific TCR from TILs based on TCR Frequency |
| **Identification and isolation of neoantigen-specific T cells/TCR from peripheral blood** | | | | | | | | |
| Lennerz  (67) | Melanoma | 1 | 1 | 5 | SIRT2; GPNMB; SNRP116; SNRPD1; RBAF600 | CD8 | / | Identify neoantigen-responsive T cells by using of MLTCs and CTLs clones to autologous tumor cell cDNA library screening |
| Cohen  (74) | Melanoma | 8 | 5 | 9 | SRPX; WDR46; AHNAK; COL18A1; ERBB2; TEAD1; NSDHL; GANAB; TRIP12 | CD8 | / | Use MHC-peptide tetramer to isolation of neoantigen specific T cells from peripheral blood |
| Gros  (75) | Melanoma | 4 | 3 | 7 | MAGEA6; PDS5A; MED13; FLNA; KIF16B; SON; KIF1BP | CD8 | / | Isolation of neoantigen specific T cells from peripheral blood based on the expression of PD-1 |
| Cafri  (76) | Epithelial cancer | 6 | 5 | 5 | SMAD5; MUC4; KRAS^G12V^; KRAS^G12D^; KRAS^G12D^ | CD4:2  CD8:3 | 9 | Isolation of neoantigen specific T cells from memory T cells subsets |
| Martin  (83) | Ovarian cancer | 1 | 1 | 5 | HSDL1; NOX4; CAPN7(Del); ZNF41; OR4C11 | CD8 | / | A library-based screening method identifies naïve, neoantigen specific T cells from small volumes of blood |

TCR, T cell receptor; TILs, tumor infiltrating lymphocytes; WES, whole-exome sequencing; ACT, adoptive cell therapy; TMG, tandem minigene; MLTCs, mixed lymphocyte-tumor cells; CTLs, cytotoxic T lymphocytes; MHC, major histocompatibility complex; PD-1, programmed cell death-1.
